# Supplementary material for: Are carbon emissions trading and green financial instruments synergistic? -Comprehensive quantitative research based on content analysis
Source: PLoS One. 2024 Mar 7;19(3):e0298601. doi: 10.1371/journal.pone.0298601 (PMC10919601; doi:10.1371/journal.pone.0298601)
Supplement: S1 Data — (ZIP) [file pone.0298601.s001.zip › Data/地级市Z_F工作报告.zip!/─■╧─/╣╠╘¡╩╨/└┤╫╘╣1⁄2╓┌║┼í╛┬φ┐╦╩2╛▌═°í┐_2013.docx]

各位代表：

　　现在，我代表市人民政府向大会作工作报告，请予审议，并请政协委员和列席人员提出意见。

　　五年工作回顾

　　过去的五年，是我市抢抓机遇、攻坚克难、转型发展、创新突破的五年。五年来，在自治区党委、政府和市委的坚强领导下，在市人大、市政协的监督支持下，我们全面落实科学发展观，凝聚全市人民的力量，共同推进工业化、城镇化、农业产业化和构建和谐社会进程，超额完成了市二届人大一次会议确定的各项目标任务。全市呈现出经济发展、民生改善、环境提升、民族团结、社会进步的良好局面。

　　——经济总量快速提升。预计2012年全市完成地区生产总值154亿元，比2007年增长83.8%，年均增长12.9%;公共财政预算总收入完成15.5亿元，比2007年增长4.2倍，其中地方公共财政预算收入完成10亿元，比2007年增长4倍;地方公共财政预算支出126.7亿元，比2007年增长3.1倍;完成全社会固定资产投资175亿元，比2007年增长3倍;实现金融存款余额232亿元、贷款余额133亿元，分别比2007年增长2.57倍和2.77倍。

　　——产业结构优化升级。三次产业结构由2007年的28:20:52调整为25:29:46。第一产业稳中求进，农业产业化步伐加快。全市粮食播种面积稳定在400万亩，粮食总产量突破80万吨。特色种养业呈规模化，创建自治区级现代生态农业示范基地13个;肉牛饲养量达到86万头，马铃薯脱毒种薯三级繁育体系基本形成，新修高标准基本农田62.5万亩，设施农业规模达到29.8万亩;加工型农业初见端倪，加工转化率达到42.8%;品牌农业效应凸显，原州区被授予中国冷凉蔬菜之乡，西吉马铃薯、芹菜荣获全国驰名商标，泾源黄牛、彭阳辣椒通过国家地理标志认证，六盘山苗木通过国家证明商标认证。2012年预计完成农业增加值38亿元，比2007年增长43.3%。第二产业实现重大突破，工业迈出实质性步伐。煤炭、岩盐、石油等资源勘探取得重大进展;煤炭产能达到510万吨，原油产量达到15万吨，电力装机容量达到770兆瓦;盐化工循环经济扶贫示范区基础设施基本完成，中铝固原煤电铝一体化项目启动，金昱元盐化工项目开工建设。2012年预计完成工业增加值25亿元，比2007年增长1.62倍。建筑业健康发展，预计实现增加值19亿元，年均增长25%。第三产业蓬勃发展，旅游业优势更加明显。六盘山旅游集团公司正式运营;修编完成旅游产业总体规划和主要景区控制性详规;实施了六盘山、须弥山、火石寨等景区开发和基础设施工程。六盘山旅游区、固原博物馆被评为国家4A级景区，红绿六盘的知名度不断扩大。西兰银综合物流园区启动建设;商贸流通、餐饮住宿等传统服务业明显提升;金融保险、中介服务、信息传媒等现代服务业快速发展。2012年预计完成第三产业增加值72亿元，比2007年增长68.5%。招商引资成效显著。创新招商引资机制，完善招商引资优惠政策，引进中铝集团、中盐集团、宁夏发电集团、福建国圣公司等一批国有企业、民营企业进入固原合作开发。五年共引进招商引资项目611个，实际到位资金163.39亿元，是前五年的5.15倍。

　　——生态移民和扶贫开发扎实推进。坚持把生态移民作为全市 “一号工程”强力推进，建成69个县内移民点、39个县外移民点，搬迁安置移民1.9万户8.23万人。其中县内9342户4.07万人，超额完成年度任务;县外9680户4.16万人，完成年度任务的61.2%。编制了《六盘山集中连片特殊困难地区扶贫攻坚规划(2011—2020年)》，扎实做好闽宁对口扶贫协作和社会帮扶，479个整村推进扶贫开发任务全面完成，减少农村贫困人口32.2万人。

　　——宁南区域中心城市暨大县城建设步伐加快。《固原市城市总体规划(2011—2030年)》已经自治区批准实施。城市建成区面积由2007年的23.8平方公里扩大到36.62平方公里。集中实施了行政办公、学校医院、星级酒店、房产商贸、市政设施等一大批重点项目;新增城市主干道54.47公里，改造主、次干道20.5公里，硬化街巷7.47万平方米，新增供排水管网71.17公里、供热管网63.6公里，新建景观休闲广场9处。大县城建设开局良好，县城框架逐步拉开;建成三营、兴隆、沙塘、泾河源、古城等一批特色小城镇。全市城镇化率由2007年的23%提高到34.85%。

　　——生态环境显著改善。建成贺家湾水源地保护、长城梁景观绿化等一批万亩以上重点生态工程，完成造林164.1万亩，退耕还林补植补造481万亩，治理水土流失面积1521.9平方公里。深入开展城乡环境综合整治，人居环境明显改善。成功创建自治区级园林城市，固原市被确定为全国生态文明示范工程试点市。

　　——重大基础设施日趋完善。六盘山机场建成通航，福银高速固原段全线贯通，青兰高速公路毛家沟至东山坡段开工建设，原州区至王洼铁路建设进展顺利，全市四级以上公路总里程达到6605公里，新修农村沥青或水泥公路5000公里，实现了乡乡通油路，81.5%的行政村通沥青或水泥路。东山坡引水、原州区东部引水、固西引水等一批重点水利工程全面完成，解决了81.5万人用水安全;宁夏中南部城乡饮水安全水源工程开工建设。建成清水河330千伏变电站，完成了一批农网、城网改造工程，率先在西北地区实现农村户户通电。全市所有行政村建成了农村信息服务站，覆盖城乡的信息网络初步形成。

　　——民生事业全面发展。五年共办理民生实事113件，民生支出占财政总支出的比例达85.8%，高于全区10个百分点。坚持优先发展教育。“两基”教育目标如期实现。高中阶段教育、学前教育、职业教育、特殊教育、民族教育健康发展。新建、改扩建固原一中、固原回中等中小学校91所，改造校舍面积122万平方米，解决了中小学校舍安全问题。义务教育阶段普惠性政策全面落实，农村学校营养改善计划全面实施。教育教学质量明显提升，初中升学率提高到95%，普通高考录取率提高到78.6%。加快科技推广应用。深入开展科技特派员创业行动，累计实施科技推广项目322项，科技对国民经济增长的贡献率达到42%，比2007年提高5个百分点。千方百计扩大就业。累计新增岗位2.66万个，新增就业4.14万人(次)，城镇登记失业率控制在4.3%以内;每年稳定转移农村劳动力26万人以上，劳务收入占农民人均纯收入的45%以上。努力增加城乡居民收入。在全区率先实施了《固原市区失地农民最低生活保障制度》;最低工资、城乡低保和医保补助标准进一步提高。预计2012年全市居民消费价格指数涨幅控制在2%左右，全市城镇居民人均可支配收入、农民人均纯收入分别达到17110元和4680元，分别比2007年增长88.4%和111%。推进医疗卫生事业发展。新建、改扩建市、县(区)医院13所、乡镇卫生院81所，村村都建有标准化卫生室，“看病难、看病贵”的问题得到初步缓解。不断完善社会保障体系。扩大高龄津贴范围，城镇职工“五险”参保覆盖面进一步扩大，养老、医疗、工伤保险等遗留问题得到妥善解决;全面启动统筹城乡居民社会养老保险和医疗保险，参保率分别达到85.57%和96.6%以上;住房公积金归集总额达到21.34亿元，贷款总额达到13.37亿元;加快保障房建设，建成廉租房、公租房、经济适用房127.69万平方米，改造危窑危房4.2万户，困难群众居住条件明显改善。推动文化固原建设取得新成效。建成乡镇综合文化站36个，精心打造了一批优秀文艺作品，原州区被命名为“中国民间文化艺术之乡”，隆德县被命名为“中国书法之乡”，西吉县被命名为“中国文学之乡”;实现了农家书屋村级全覆盖，完成直播卫星公共服务户户通工程25.39万户，广播电视综合覆盖率达到98%;举办了两届全国群众登山健身大会暨宁夏六盘山登山节，全民健身活动广泛开展。

　　——社会建设和谐稳定。成功举办建市10周年庆祝活动，充分展示了全市经济社会发展的辉煌成就，极大地激发了全市干部群众干事创业的热情。深入开展进一步营造风清气正发展环境和民风建设活动，发展环境进一步优化。顺利通过 “五五”普法验收，“六五”普法全面启动。推进“平安固原”建设，严厉打击各类刑事犯罪，群众安全感不断提升。深入开展矛盾纠纷和不稳定因素排查化解。推行领导干部大接访制度，一些信访积案得到化解，群众合法合理诉求得到妥善解决。完善应急管理体系，突发公共事件得到及时有效处置。集中开展道路交通、食品药品等领域的安全整治活动，安全生产形势总体平稳，食品药品安全良好。城乡社区建设有序推进，基层民主建设得到加强。依法加强宗教事务管理，民族团结、宗教和顺的局面进一步巩固。启动自治区“双拥模范城市”创建活动，国防动员教育不断加强。人口和计划生育工作成效明显，少生快富“硝沟模式”在全区推广，预计2012年全市人口自然增长率为10.32‰，比2007年下降0.92个千分点。统计、档案、气象、地震、人防、外事侨务、地方志、残疾人等事业取得新的进步。

　　——政府自身建设不断加强。坚持简政放权，规范行政审批事项182项，取消行政审批事项19项。制定完善决策议事制度，政府科学民主决策水平不断提升。全面完成政府机构改革，积极推进事业单位改革。推行政务公开，完善三级便民服务体系，办事效率明显提高。深入开展学习实践科学发展观、“思想大解放、树立新形象”等活动，认真学习贯彻党的十八大精神、自治区第十一次党代会和市第三次党代会精神，干部队伍的思想观念明显转变。坚持依法行政，累计办理人大代表议案、建议386件，政协委员提案1429件，办复率均为100%。认真落实党风廉政建设责任制，强化行政效能监察、审计监督和责任追究，不断推进政府廉政建设。

　　各位代表，五年的团结拼搏，我们取得了经济社会、城乡面貌、人民生活条件、干部工作作风明显变化的可喜成绩。五年的不懈努力，我们办成了一批期盼已久的民生实事、事关长远发展的大事。取得这些成就，靠的是自治区党委、政府和市委的坚强领导，靠的是自治区厅局及社会各界的大力支持，靠的是全市人民的团结奋斗、艰苦创业。在此，我代表市人民政府，向辛勤工作在各条战线上的广大干部职工，向给予政府工作大力支持的人大代表、政协委员、各民主党派、工商联、无党派人士和各人民团体，向中央、自治区驻固单位和驻固部队、武警消防官兵，向为固原发展进步做出贡献的老同志，向关心、支持固原改革发展稳定的各级组织、各界人士、全市广大人民群众，表示衷心的感谢!

　　五年的生动实践，积累了宝贵的经验。一是坚持党的领导，创造性地贯彻落实好中央、自治区和市委的决策部署是做好政府工作的基本前提;二是坚持科学发展，推进工业化、城镇化和农业产业化是提升综合实力的基本出路;三是坚持解放思想，争政策、争项目、争资金和扩大招商引资是加快脱贫致富、增强发展后劲的基本途径;四是坚持以人为本，保障和改善民生是做好一切工作的基本目的;五是坚持求真务实，不断凝心聚力、开拓创新是推动各项事业蓬勃发展的基本动力。

　　实现到2020年与全区同步进入全面小康社会是我们矢志不渝的政治任务，但面临的困难大、问题多，具体表现为“四低三难”。“四低”：一是小康实现程度低，尤其是人均GDP低。全市小康社会实现程度2012年预计为65%，分别比全国、全区低20和8个百分点，人均GDP比全国、全区平均水平分别低26527元和23836元。二是城镇化率低。全市城镇化率分别比全国、全区平均水平低18.52和17.05个百分点，城镇集聚辐射带动能力不强。三是城乡居民收入低。城镇居民人均可支配收入比全国、全区平均水平分别低6931元和2700元，农民人均纯收入比全国、全区平均水平分别低2933元和1366元。四是群众受教育程度低。全市人口平均受教育年限为7.27年，距离全面小康目标还有3.23年。“三难”：一是控制人口增长难。人口自然增长率分别比全国、全区高6.15和1.97个千分点，因政策等原因人口出生处于刚性增长期，短期内难以降低人口出生水平。二是推进扶贫攻坚难。目前全市还有50.1万贫困人口，贫困程度深，贫困面广，消除贫困任务艰巨。三是提升发展环境难。一方面，生态环境脆弱，水资源匮乏，交通等基础设施滞后，发展的硬环境亟待改善;另一方面，干部群众思想解放的深度不够，等靠要的观念需要转变，个别干部的能力和作风与新形势新任务的要求还不相适应，发展的软环境更需优化。

　　各位代表，在责任和困难面前，我们必须要有坐不住的紧迫感，慢不得的责任感，误不起的使命感，变压力为动力，化挑战为机遇，解放思想，攻坚克难，奋勇前进!

　　五年奋斗目标和任务

　　经过五年的发展，我们站在了跨越发展、强市富民的新起点，踏上了与全区同步进入全面小康社会的新征程。未来五年的快速发展有大环境支撑，大机遇支援，大政策支持。一是我国发展仍处于大有作为的战略机遇期，确保到2020年实现全面建成小康社会宏伟目标能给我们创造跨越发展的大环境。二是国家支持西部地区、老少边穷地区和我区发展的政策力度会加大，给我市实现强市富民带来大机遇。三是我区实施的沿黄经济区、百万人口扶贫攻坚战略和“两区”建设，牵引我市快发展。四是自治区支持脱贫致富的政策、项目、资金等助推我市大发展。五是我市多年发展形成的资源、人文、环境等优势蓄势待发，潜力巨大。

　　根据市第三次党代会战略部署，今后五年，政府工作总体要求是：高举中国特色社会主义伟大旗帜，以邓小平理论和“三个代表”重要思想、科学发展观为指导，深入贯彻落实党的十八大、自治区第十一次党代会精神，以抓“把握一个主题，推动三个转变，实施六大战略”为主旨，以抓产业富市、生态立市、依法治市为统领，以抓教育、计生、水利为基础，以抓基础设施、民生改善、社会管理、改革开放为重点，以抓规划引领、争发展政策、争项目资金、招商引资、优化环境为措施，不断提升特色产业竞争力、中心城市吸引力、生态环境承载力、文化旅游影响力、社会和谐凝聚力，打造六盘山片区“四化”同步示范区和西部生态文化旅游城市，为建设新固原，与全区同步进入全面小康社会打下决定性的基础。(简称“五抓五提升”)

　　总体目标是：到2017年全市小康社会实现程度达到80%以上，其中，经济发展达到75%以上、社会和谐和生活质量均达到90%以上、民主法制达到95%以上、文化教育和资源环境均达到73%以上;到2020年实现程度达到95%以上，基本与全区同步进入全面小康社会。具体目标是：

　　——实现三个翻番：地区生产总值、固定资产投资、公共财政预算收入比2012年翻1.5番，2017年分别超过400亿元(现价)、500亿元、30亿元，年均分别增长14%、28%、25%。

　　——实现三个倍增：累计新增就业岗位、城镇居民人均可支配收入、农民人均纯收入实现倍增。2017年分别达到15000个(五年累计5.5万个)、34200元和10000元以上。

　　——实现三个提升：工业化、城镇化、农业产业化水平显著提升。工业占GDP比重达到30%，城镇化率达到50%，农产品加工转化率达到50%以上。

　　——实现三个确保：确保完成25万生态移民和40万贫困人口脱贫任务;确保提高城乡居民的社会保障水平;确保人口自然增长率控制在9‰以内。

　　——形成三大产业基地：产值超过200亿元的冶金和盐化工产业基地，产值超过100亿元的煤电油能源产业基地，产值超过50亿元的宁南绿色有机农副产品加工集散基地。

　　——打造三个经济圈：以固原为中心，辐射半径500公里的旅游休闲度假圈、300公里的商贸物流圈、100公里的购物生活圈。

　　——建成三个示范区：国家级扶贫开发示范区、全国民族团结和谐示范区、西部地区生态文明示范区。

　　——创建三个国家级城市：国家生态园林城市、国家卫生城市、国家环保模范城市。

　　各位代表，今后五年是我市全面建设小康社会的决定性阶段，实现与全区同步进入全面小康社会的任务十分艰巨。只要我们正视困难，用好机遇，发挥优势，坚定信心，不动摇、不懈怠、不折腾，就能誓拔穷根，迈向小康。

　　今后五年的主要工作任务：

　　一、增收致富奔小康，全力以赴打赢移民和扶贫两场硬仗

　　脱贫致富是全面建设小康社会的核心，生态移民和扶贫攻坚战略是实现50万贫困人口过上美好日子的主抓手。

　　全面完成生态移民工程。按照生态移民规划和“搬得出、稳得住，逐步能致富”的要求，积极推行“头营移民模式”，依托中心集镇建设移民大社区，统筹推进社区建设、公共服务、产业培育、社会管理，提前全面完成“十二五”生态移民任务。引导移民群众转变思想观念和生产生活方式，依靠组织帮助和自我诚实劳动，率先致富奔小康。积极争取提前实施“十三五”移民任务，争取再搬5—10万人。

　　大力推进扶贫开发工程。以《六盘山集中连片特殊困难地区扶贫攻坚规划(2011—2020年)》为指导，创新扶贫开发机制，整合各类扶贫资源，坚持产业扶贫与项目扶持相结合，技术支持与技能提高相结合，集中连片开发与新农村建设相结合，支持能人率先发展与济困救弱相结合，闽宁协作等社会帮扶与自力更生相结合，落实“四到”扶贫措施，大幅减少贫困人口。2017年，绝对贫困人口控制在10万人以内。

　　重点实施增收致富工程。以提高收入为核心，逐步改善人民的生活质量。认真落实强农惠农政策，增加群众转移性收入。强化技能培训，发展特色产业，增加经营性收入。盘活农村土地、房屋、林权等资源，增加财产性收入。壮大规范劳务中介组织，加大技能培训力度，保障务工人员权益，加快农村劳动力转移，年转移就业30万人以上，人均年创劳务收入1万元以上，增加工资性收入。

　　二、引企入园育龙头，坚定不移做强做大工业经济

　　工业是强市富民的希望和跨越发展的引擎。市财政每年安排工业发展资金3000万元，支持实施“工业强市”战略。以园区为载体，以项目为支撑，以招商为抓手，坚持循环发展、绿色发展理念，不断提升工业化水平。2017年，工业增加值达到120亿元以上，年均增长25%。

　　创新机制，发展产业园区。用好政策，理顺管理，全力推进盐化工循环经济扶贫示范区建设，发展以电解铝为主的冶金产业和以盐化工为主的化工产业。规划建设轻工产业园，发展以电子产品、农副产品为主的加工业。支持县(区)发展优势特色产业园。探索市、县共建产业园区机制(产值、税收、就业、招商任务等属于引进的县、区)，解决县(区)发展工业成本高、代价大、配套弱、规模小等问题，避免产业雷同、重复建设、影响生态，实现产业集群发展。

　　培育主导产业和龙头企业。进一步加快资源勘探工作。引进大企业、大集团参与合作开发，形成产值过200亿元的冶金和盐化工产业，争取建成年产100万吨电解铝及配套项目，金昱元32万吨离子膜烧碱、40万吨PVC树脂、100万吨电石渣制水泥项目，中盐集团100万吨真空制盐等项目;形成产值过100亿元的能源产业，建成王洼千万吨级矿区、彭阳60万吨原油开采项目、新增电力装机容量1000兆瓦;形成产值过50亿元的特色农产品加工业;发展战略性新兴产业，建成一批风力发电、光伏光热项目。大力培育和引进龙头企业10家，带动工业与信息融合，促进技术与市场结合，加快产业发展升级。支持企业争创名牌产品、驰名商标。组建中小企业服务中心，支持发展中小微科技型企业，促进民营经济快发展、大提升。

　　完善政策，扩大招商引资。抢抓自治区建设内陆开放型经济试验区和银川综合保税区的新机遇，准确定位产业，科学布局园区，制定特殊政策，依托中阿博览会、闽宁对口协作等平台招大商，在引进中央大型企业和民营骨干企业上取得新进展。招商引资到位资金年均增长30%以上，五年累计达到600亿元以上。

　　三、以工促农创品牌，持之以恒做实做精生态农业

　　因地制宜、因势利导，发展生态农业是我们坚持的方向。通过调结构、建基地、抓龙头、创品牌，推进特色农业增效战略。2017年，农业增加值达到80亿元，年均增长6%以上。

　　突出发展特色优势产业带。建成清水河流域冷凉蔬菜、枸杞产业带;葫芦河流域马铃薯、西芹产业带;渝河流域中药材、花卉产业带;泾河流域苗木、黄牛养殖产业带;红河、茹河流域辣椒、经果林产业带。以发展养牛大户、示范村、示范乡镇为抓手，建成百万头肉牛繁育养殖基地;以推广新品种、新技术为抓手，建成百万亩优质马铃薯种薯推广种植基地(生产原原种2.5亿粒，种薯繁育面积达到30万亩);以蓄水、覆膜为保障，以建“菜篮子”、“米袋子”为要求，建成二百万亩旱作节水农业基地，设施农业达到30万亩。

　　扶持龙头企业，健全营销网络。创新扶持政策，培育年销售收入过6000万元的国家级龙头企业5家，年销售收入过1000万元的自治区级龙头企业50家，年销售收入过500万元的市级龙头企业150家。培育壮大农村专业合作组织，发展订单农业。健全农产品市场营销体系，建成3个年交易额上亿元的农产品综合交易市场、18个专业批发市场、15个乡镇农贸市场。

　　争创六盘山绿色有机品牌。建立健全农产品质量安全监管体系，积极开展农产品产地认定、产品认证，推进农业标准化建设。巩固提升“六盘山”品牌，争创绿色有机品牌，培育国内知名农产品品牌5个、自治区级知名农产品品牌25个，提高农产品附加值和市场竞争力。

　　四、创新机制强基础，毫不动摇做活做优现代服务业

　　文化旅游业是固原经济发展的优势和潜力所在。市财政每年安排1000万元，推进文化旅游品牌战略，提升服务业质量。2017年，第三产业增加值达到170亿元，年均增长14%。

　　重点发展文化旅游业。深度挖掘地域特色文化，争取申请历史文化遗产保护。深入推进公民道德建设和加快实施文化惠民工程，建设一批公益性文化项目，实现公共文化服务设施向社会免费开放。加快文化产业发展，促进文化与旅游产业相融合，实施一批重大文化旅游产业项目，组建文化团队，创作一批文化艺术精品节目，发挥文化引领风尚、教育人民、服务社会、推动发展的作用，引导公民自觉履行法定义务、社会责任和家庭责任。培育文化旅游名企、名人。发挥六盘山旅游集团公司的作用，加大宣传营销力度，打造须弥山—火石寨—六盘山—崆峒山旅游精品线路。把六盘山国家森林公园、须弥山建成5A级旅游景区，增强对国内外游客休闲度假的吸引力。2017年，旅游社会总收入突破30亿元。

　　加快发展新型服务业。建成西兰银综合物流园区和各县城综合物流中心，培育年销售收入上亿元的物流企业12家，形成西部重要的物流集散地。加大土地收储，加快发展房地产和工业地产。积极发展电子商务、中介服务、信息咨询、社区服务等新型业态。优化金融环境，支持各类银行、证券公司等金融机构在固原设立分支机构，支持地方经济发展。鼓励发展小额信贷公司、村镇银行和融资平台、担保机构，激活社会投资，拓宽融资渠道。

　　巩固提升传统服务业。引进大企业、大集团发展连锁经营，打造高端精品商业区。改造提升餐饮住宿、商贸物流、交通运输等传统服务业。培育地方特色品牌的名店、名小吃、农家乐，提升民族特色餐饮业整体水平。全社会消费品零售总额年均增长15%。

　　五、产城一体促统筹，齐心协力建设生态文化旅游城市

　　产业是做大城市的根基，城市是推动产业升级的孵化器，推进城镇化是我市经济发展新的增长点。坚持中心城市带动战略，统筹发展产业、城市、生态，做靓做特宁南城镇集群。

　　坚持规划引领城镇布局。严格执行城市规划，依照城市总规，编制控规、详规和专项规划，引导中心城市、大县城、小城镇合理分工、功能互补、特色发展。高标准建设、高水平管理，推进区域中心城市转型发展，彰显魅力。指导支持各县(区)按照规划推进大县城、18个中心镇和一批新农村建设，增强产业发展、公共服务、吸纳就业、人口集聚功能。2017年，城市区人口达到32万人，建成区面积达到50平方公里。

　　坚持基础设施先行。加快完善新区道路、供排水、供电、供气等基础设施，建成九龙路，打通新区与西南新区的大通道。改造完善老城区功能，配套建设休闲广场、停车场、垃圾中转站和公厕。以固原博物馆为中心，建设体现固原古城风貌的特色建筑群。加大城中村改造，基本消除城市棚户区。新修东环线，完成国道309线市区过境段、北环路建设，形成畅通快捷的外环路。争取实现福银高速市区段向南改线，拓展城市发展空间。

　　坚持城市发展定位。按照建设“立足宁南、辐射周边、对接三省”的生态文化旅游城市，切实提升区域中心城市吸引力。老城区以商贸、餐饮、居住为主，新区以文化、教育、行政为主，西南新区以物流、生产性服务业、居住为主，西部新区以工业为主，形成功能合理的城市布局。

　　坚持壮大县域经济。引导支持各县(区)发挥优势，利用低丘缓坡等未利用地，发展产业，建设城市。把原州区打造成以设施农业、新型工业、现代服务业为主的宁南区域中心城市核心区，把西吉县打造成以马铃薯产业为主的固原向西开放的桥头堡，把隆德县打造成以中药材、文化旅游业为主的国家生态补偿示范县，把泾源县打造成以苗木、养牛、生态旅游业为主的国家生态和水资源补偿示范县，把彭阳县打造成以能源化工产业为主的宁南县域经济排头兵和固原向东开放的桥头堡，逐步形成产业特色突出、城乡统筹发展的县域经济新格局。

　　六、善谋多争抓落实，鼓足干劲以大项目带动大发展

　　项目是我市经济发展的第一推动力。争取上一批产业项目、基础项目、民生项目，支撑固原经济社会科学发展。五年累计完成固定资产投资近2000亿元(其中重大项目见附件一)。

　　重视生态环境项目建设，创建国家环保模范城市。以争取国家级生态补偿示范区为载体，将六盘山核心区纳入国家生态资源和水资源补偿试点范围。推进大六盘生态经济圈建设，巩固扩大退耕还林和禁牧封育成果，实施水源涵养林、农田防护林、特色经济林、城镇景观生态林建设工程，新造水源涵养林100万亩，封育50万亩，城乡环境绿化10万亩，构筑西部地区重要生态安全屏障。规划建设清水河湿地公园、古雁岭城市休闲主题公园和长城梁、东岳山等一批生态文化园，创建“城中有山、山中有景”的国家生态园林城市。2017年，全市森林覆盖率达到25%以上。治理水土流失面积1000平方公里，治理程度达到85%以上。加强环境保护，全面完成自治区下达的节能减排任务。以治脏治乱治差、绿化美化亮化为重点，持续开展城乡环境综合整治，创建国家环保模范城市和卫生城市。

　　加强水利项目建设，提升供水保障能力。以水资源综合开发利用为重点，加快库坝窖池联调工程建设，实现水资源高效配置，推进节水型社会建设。建成宁夏中南部城乡饮水安全水源工程，同步建设配水工程，解决我市100万人吃水难问题。兴建扬黄扩灌固原市区延伸工程，实现引泾入城和引黄入城用水安全双保障。

　　加强交通项目建设，提升城市辐射力。加快构建区域中心交通枢纽，建成兰青高速毛家沟至东山坡段、固原至西吉一级公路、西吉至静宁一级公路、青石嘴至彭阳一级公路、福银高速泾源出口至县城一级公路。争取建设宝中铁路复线、灵武鸳鸯湖至平凉高速公路原州区和彭阳过境段、固原经彭阳至麻子沟圈高速公路，打通固原与周边地区的高速通道。改造提升县乡村道路等级，实现县县通一级公路、乡镇通三级以上公路、行政村通沥青或水泥路。争取改扩建固原火车站和三营火车站。实施城乡客运一体化项目，改革市区公共交通管理体制，发展政府主导的市区公共交通。加快天然气入户工程，到2017年，城市居民户户通天然气。全面改造升级城乡电网，提高电力保障能力。推进“数字固原”建设，提升信息化水平。

　　七、民生优先铸和谐，坚持不懈加强和创新社会建设

　　保障和改善民生是政府一切工作的出发点和落脚点。多谋民生之利，多解民生之忧，多办民生实事，努力让人民过上更加美好的生活，是我们工作的目标。

　　办好人民满意的教育。研究出台固原市办人民满意教育的实施意见。进一步提升基础教育，基本普及高中阶段教育，大力发展职业教育，关心支持特殊教育，促进教育协调发展。重视发展学前教育，每个乡镇至少建1所公办标准化幼儿园，普及学前三年教育。优化教育布局和资源配置，大力改善办学条件，鼓励名校跨区域办分校。改善教学设备，加大师资培训，提升教育教学质量，让更多的学生上好学。大力培养和发展校园文化，加强青少年活动场所建设，重视青少年思想道德和素质教育。加快科技创新、推广和应用，科技进步对国民经济增长的贡献率达到50%。支持引进教育、卫生、科研等方面的专业技术人才。

　　健全就业和社保体系，提高收入水平。完善就业创业服务体系，以产业园区带就业，以创业平台促就业，以公益投入保就业，以技能培训推就业，力争家家有人稳定就业。城镇登记失业率控制在4.5%以内。完善社会保障体系，实现城乡居民基本养老保险、基本医疗保险全覆盖，健全以城乡最低生活保障为主的新型社会救助体系，实现扩面提标。加快保障性住房建设，改善群众居住条件。办好慈善产业园，发展慈善事业。完善低收入群体价格补贴与物价上涨联动机制，保持物价总水平基本稳定。创造条件，争取政策，不断提高干部职工和城乡居民收入。

　　提高人民健康水平。坚持保基本、强基层、建机制，健全农村三级医疗卫生服务网络和城市社区卫生服务体系，加强医疗卫生事业和队伍建设，为群众提供安全有效、方便价廉的公共卫生和基本医疗服务，让大病不出城市，小病不出社区。加强食品药品监管，保障饮食用药安全。坚持计划生育基本国策不动摇，降低生育水平，切实提高出生人口素质。

　　加强和创新社会管理，维护社会稳定。牢固树立“团结稳定是福、分裂动乱是祸”的思想。按照党委领导、政府负责、社会协同、公众参与、法制保障的社会管理模式，推进和谐社会建设。加强城乡社区建设，提高社区管理服务水平。扎实推进“六五”普法，加快依法治市进程。健全社会治安防控体系，有效防范和打击违法犯罪活动。畅通和规范群众诉求渠道，全面加强人民调解组织建设，认真解决好涉及群众切身利益的矛盾和问题。严格落实安全生产责任制，加强重点行业领域安全生产专项整治，最大限度地减少安全事故。完善应急管理机制，提高应对突发公共事件的能力。尽快开展地震活断层探测工作，为建设重大工程和市民居住安全提供科学依据。积极推进民族团结进步事业，促进各民族和睦相处、和衷共济、和谐发展。依法加强宗教事务规范化管理，维护宗教领域和谐稳定。深入开展国防动员教育，推进“双拥模范城市”创建活动。推动基层民主建设，加强公民诚信和文明意识教育，培育自尊自信、理性和平、积极向上的社会心态。以加强人口、住房“两实”信息管理为内容，完成违章建房清理和合法建房产权登记工作。

　　进一步深化改革，扩大开放。抢抓“两区”建设新机遇，在用足用活国家和自治区支持固原各项政策的同时，制定我市落实“两区”建设实施方案，争取特殊政策、重大项目支持，形成政策洼地、投资热土。认真落实国家和自治区出台的改革方案。积极争取实行扩权强县、强镇试点，形成权责利、人财事相协调的运行体制机制。积极推进户籍制度、农村房屋产权制度改革。改进规划、土地、项目、资金等行政审批制度。理顺市、区城市管理机制，提高城市管理水平。协调推进财税金融体制改革。积极稳妥推进土地承包经营权流转。稳步推进事业单位改革，加快文化、教育、科技、医药卫生体制改革，促进公共资源均衡配置。加快建立对外开放、宣传，对内放开、搞活的新机制。对外把我市作为宁夏走出去开展经贸合作、请进来聚集生产要素的南大门，作为展示、宣传宁夏的新窗口定位发展。扩大开放旅游、矿产等优势资源和特许经营权，吸引大企业来固合作发展、壮大实体经济;对内进一步放开民营企业、个体工商户参与市场竞争的领域，搞活区域经济。

　　八、依法行政树形象，尽心竭力建设人民满意政府

　　建设责任政府、法治政府、服务政府、廉洁政府是实现与全区同步进入全面小康社会的重要保障。

　　解放思想，创新发展。坚持把学习贯彻党的十八大精神贯穿政府工作始终，认真贯彻执行自治区党委、政府以及市委的一系列决策部署。进一步解放思想，“面向全国看固原，放眼未来看固原”。通过培训学习，思想教育，观摩交流，外出考察，考核奖惩，激发政府班子及组成人员干事创业的激情，提高研究政策、谋划项目、推进发展、改善民生、维护稳定、抓好落实的能力和水平。

　　改进作风，助推发展。加快推进政府职能以审批管理为主向以监管服务为主转变，减少审批事项，优化办事流程，推行政务公开，提高办事效率。以每年实施一批事关经济社会发展的大事和改善民生的实事为重点，明确县(区)、部门抓发展、惠民生、保稳定、争政策、争项目、争资金、招大商、奔小康的责任，从市政府班子成员做起，带头抓落实。认真贯彻执行中央“八项”规定，进一步改进工作作风，加强调查研究，密切联系群众，解决实际问题;坚持实干富民，实干兴邦，勇于开拓，敢于担当，多干让人民满意、得实惠的好事实事;进一步改进文风会风，少开会、开短会，讲短话、真话、实话;加强政风行风建设，完善行政问责机制，整治庸懒散软奢等不良风气，提高政府公信力。

　　优化环境，促进发展。进一步深化营造风清气正发展环境活动，建立完善长效机制，切实提高政府效能。全面推行限时办结制、服务承诺制、效能告诫制等制度，从制度上保证服务质量。对重大项目、重点工程实行集中联审联办，营造亲商、敬商、重商、护商的浓厚氛围，依法保护企业经营者和外来投资者的合法权益。加强政务、商务、社会诚信和司法公信建设，在全社会形成良好的发展环境。

　　依法行政，保障发展。加强干部法制教育，提高依法行政意识和水平。认真执行人大及其常委会的决议，自觉接受监督。主动加强与人民政协的联系，认真听取各民主党派、工商联、无党派人士和人民团体的意见。自觉接受社会监督、舆论监督，发挥媒体在推进工作中的积极作用。坚持与人大代表、政协委员的联系制度，及时、高质量地办理议案、建议和提案。支持法院、检察院的基础设施建设。完善重大决策专家咨询论证和社会公示听证制度，提高政府科学民主决策水平。成立规划审批委员会、土地决策委员会和财经领导小组，加强和规范规划审批、土地管理、资金安排等工作。公开工程招投标、经营性土地出让、政府采购等公共资源交易内容，确保行政权力在阳光下运行。严格执行廉政建设责任制，履行好“一岗双责”，做到干部清正，政府清廉。

　　各位代表，长征精神砺雄志，六盘儿女竞风流;小康宏图聚民力，幸福固原展新姿。把握大好形势，拿下艰巨任务，我们必须以超常的思维谋划发展，以超常的速度跨越发展，以超常的举措推动发展。只要我们一切为了群众，紧紧依靠群众，顽强奋斗，艰苦奋斗，不懈奋斗，建设小康社会的目标一定能够实现!

　　2013年主要工作

　　2013年开好局、起好步，对今后五年工作至关重要。按照市委三届三次会议提出的“五加快五统筹”的总要求，以抓好100个重点项目和10件大事、15件实事(分别见计划报告附件和政府报告附件二、附件三)为重点，以“七抓七促”推动政府工作上水平。

　　一、抓协调督查，促健康发展。全年计划实现地区生产总值增长14%，达到176亿元;固定资产投资增长30%，达到228亿元;地方公共财政预算收入增长20%，达到12亿元;城镇居民人均可支配收入增长13%，达到19330元;农民人均纯收入增长14%，达到5335元。居民消费价格指数涨幅控制在3.5%以内;城镇登记失业率控制在4%以内;人口自然增长率控制在11‰以内;全面完成自治区下达的节能减排任务。加强对经济运行的监测分析，对投资及重点项目的协调督查，切实提高经济发展的质量和效益。

　　二、抓生态移民，促扶贫攻坚。完成县内移民8735户38597人，提前完成“十二五”县内移民任务。继续加强与县外移民接收市、县(区、市)的对接，争取完成县外移民8556户37330人。积极解决好安置移民增收致富问题，把移民新村建成团结村、和谐村、小康村。切实做好移民迁出区退耕造林等生态恢复工作。整合4.5亿元资金，吸引金融部门参与支持新一轮315个贫困村整村推进扶贫开发工程;新增互助资金2000多万元，吸引信贷资金投入1亿元以上，推进产业和项目扶贫;加强闽宁协作、社会帮扶工作，减少贫困人口7.5万人。

　　三、抓重点项目，促投资增长。利用“冬闲”，加快项目前期工作，争取早日开工固原至西吉一级公路、西吉至静宁一级公路、中庄水库、天然气入户工程、保障性住房、固原市人民医院、固原二中扩建、清水河湿地公园等100个重点建设项目。积极争取国家、自治区项目补助资金30亿元，实现融资5亿元(市本级)。抓好招商引资工作，年内引进实际到位资金70亿元以上。争取自治区召开第五次固原工作会议，出台支持固原与全区同步进入全面小康社会的特殊政策，布局重大项目，推动我市跨越发展。

　　四、抓园区建设，促产业升级。完成盐化工循环经济扶贫示范区基础设施建设，全力推进中铝一期电解铝、金昱元盐化工、中盐集团真空制盐、天然气综合利用等项目前期工作，力争早日开工建设。启动西部新区轻工业园基础设施和标准化厂房建设。加快王洼千万吨级矿区建设进度。支持县(区)特色产业园及项目建设。新建种植业产业示范园60个，建设种薯繁育基地25万亩，标准化百头以上肉牛养殖园区20个，新建设施农业2万亩，每个县(区)新引进或发展1家投资5000万元以上农业产业化龙头企业。全面完成六盘山长征景区、西吉火石寨、泾源老龙潭旅游基础设施建设，办好大型节会，全年接待游客220万人次以上，实现旅游社会总收入10亿元以上;完成西兰银综合物流园区 “七通一平”基础配套和四大功能区的主体工程建设。

　　五、抓基础设施，促城市建设。加快区域中心城市建设，完成控制性详细规划和专项规划。开工建设东环线，建成九龙路、北环路，拉开城市框架。加快市区限价房、城市规划展示馆、固原博物馆中心广场应急避难场所等重点项目建设。启动自治区卫生城市和国家生态园林城市创建活动。加快大县城建设。每个县(区)规划建设1—2个特色鲜明、辐射带动力强的中心镇。继续开展城乡环境综合整治行动，着力对城乡结合部、重点集镇、大村庄和国省道沿线环境进行整治，建立长效保洁机制，提高规范化管理水平。集中开展违法建筑治理活动。

　　六、抓民生实事，促社会稳定。办好事关民生的15件实事，促进教育、科技、就业、医疗、文化、计生等事业发展。新建40个城乡标准化社区，提升社区服务功能。落实信访工作责任制，及时解决群众合法合理诉求。开展道路交通等安全生产专项整治活动，确保社会安全稳定。完善城市综合监控报警系统建设，打击各类违法活动，切实提高社会管控水平。建成固原市应急指挥平台，提高应急处置能力。实施“少生快富”3000例，出生人口政策符合率达到86%以上。加强国防动员和民兵预备役建设。全面贯彻党的民族宗教政策，推进宗教事务规范化管理。充分支持统计、档案、气象、地震、人防、外事侨务、地方志、残疾人等单位发挥在促进科学决策、社会和谐、经济发展、文化传承等方面的作用。

　　七、抓作风转变，促自身建设。把认真学习贯彻落实党的十八大精神作为首要政治任务，不断武装头脑、学以致用、推动工作。进一步弘扬民主、务实作风，建立健全重大事项集体决策制、重点工作分工负责制、重点项目跟踪督办制和行政问责制。认真开展“作风建设推进年”活动，落实好中央“八项”规定和市委关于改进作风的要求，进一步深入基层，深入一线，研究解决工作中存在的困难和问题。大力倡导雷厉风行、务实高效的工作作风。积极营造敢干事、会干事、干成事、不出事的良好氛围，以良好的形象，赢得群众的信任、支持和拥护。

　　各位代表!回顾昨天，优异的成绩令人鼓舞，我们要倍加珍惜;放眼今天，六盘大地生机盎然，我们要倍加珍重;展望明天，新固原的锦绣画卷徐徐展开，我们要倍加珍爱。拔穷根、奔小康是组织重托、人民期盼，快发展、惠民生是政府职责、人民要求。让我们在自治区党委、政府和市委的坚强领导下，以党的十八大精神为指引，奋发有为，务实苦干，为建设新固原、开创与全区同步实现全面小康社会的新局面而努力奋斗!
